# Steamed panax notoginseng mitigates CA-MRSA USA300-induced necroptosis in human neutrophils

**DOI:** 10.3389/fphar.2025.1546652

**Published:** 2025-05-30

**Authors:** Lulu Zhang, Xiaoyu Feng, Hongsa An, Weifeng Yang, Yuwen Xia, Bo Wen, Haoran Zheng, Yihuan Chen, Yungchi Cheng, Chunyan Jiang, Cheng Lu, Yong Tan

**Affiliations:** ^1^ Institute of Basic Research in Clinical Medicine, China Academy of Chinese Medical Sciences, Beijing, China; ^2^ Medical Experimental Center, China Academy of Chinese Medical Sciences, Beijing, China; ^3^ Department of Pharmacology, Yale University School of Medicine, New Haven, CT, United States; ^4^ Dermatological Department, Beijing Hospital of Traditional Chinese Medicine, Capital Medical University, Beijing, China

**Keywords:** steamed Panax notoginseng, CA-MRSA, virulence factors, polymorphonuclear neutrophils, necroptosis

## Abstract

**Background:**

Community-associated methicillin-resistant *Staphylococcus aureus* (CA-MRSA) disrupts innate immunity by inducing necroptosis in polymorphonuclear neutrophils (PMNs), a process linked to excessive inflammation and tissue damage. CA-MRSA releases virulence factors that enhance its pathogenicity by disrupting the host’s innate immune response, particularly impairing the phagocytic function of PMNs. Steamed Panax notoginseng (S-PN), a traditional Chinese medicine (TCM), has demonstrated immune-regulatory and anti-inflammatory properties, showing promising therapeutic effects in alleviating the severe inflammatory responses induced by pathogenic microbial infections.

**Objective:**

This study aims to investigate the pharmacological effects and mechanisms of S-PN alleviating CA-MRSA-induced PMN necroptosis by suppressing MRSA virulence factors and inhibiting the RIPK1/RIPK3/MLKL signaling pathway, thereby attenuating inflammatory damage.

**Methods:**

A co-culture model of MRSA USA300 strain and PMNs isolated from healthy human blood was established to observe the changes in necroptosis marker HMGB1, PMNs counts, ROS, chemokine MCP-1 and pro-inflammatory cytokines IL-1β, IL-8, TNF-α. RNA-seq was employed to analyze the effects of S-PN on the transcriptional expression of pathogenesis-related genes of MRSA. RT-PCR was utilized to validate the expression of S-PN on MRSA virulence factors and PMNs necroptosis related genes.

**Results:**

S-PN significantly inhibited HMGB1, ROS, MCP-1, IL-1β and IL-8 in MRSA-PMN co-cultures, the PMN count in the S-PN group was higher than that in the model group. S-PN downregulated MRSA pathogenic-associated *S. aureus* infection and quorum sensing signaling pathways, and significantly reduced the virulence factors PSM and PVL. S-PN suppressed the expression of genes associated with necroptosis ripk1, ripk3, and mlkl in PMNs.

**Conclusion:**

S-PN alleviates CA-MRSA infection-induced immune damage through dual mechanisms: suppression of bacterial virulence factors (PSM and PVL) and inhibition of PMNs necroptosis. These findings underscore its potential as a complementary therapeutic strategy against CA-MRSA infections, providing a theoretical foundation for integrating TCM into adjuvant treatments for drug-resistant bacterial infections.

## 1 Introduction

Community-acquired methicillin-resistant *Staphylococcus aureus* (MRSA) infections pose a significant threat to global public health (Palavecino, 2020). The incidence of CA-MRSA infections has been rising annually since the 1990s, with USA300 being the predominant strain in the United States (Ahmad-Mansour et al., 2021). CA-MRSA virulence factors, such as Panton-Valentine leukocidin (PVL), α-hemolysin (Hla), and phenol-soluble modulins (PSM), are critical to its pathogenicity and contribute to the impairment of innate immune responses (Tenover and Goering, 2009). The emergence of antibiotic resistance has further complicated the treatment of CA-MRSA, necessitate novel therapeutic strategies in clinical anti-infection research (Peacock and Paterson, 2015). Treatment that targets immune damage is an important part of the fight against MRSA infection.

MRSA can undermine the immune defense capacity of the host by disrupting the immune responses of immune cells (Chen et al., 2023). Clinical studies have shown that functional defects in polymorphonuclear neutrophils (PMNs) are closely associated with recurrent infections (Rungelrath and DeLeo, 2021; Warheit-Niemi et al., 2022). PMNs, as the first line of defense in innate immunity, are typically the first responders during MRSA infection, engaging in phagocytosis to eliminate the pathogen (Rungelrath and DeLeo, 2021). One of the key strategies by which MRSA disrupts the host immune response is by inducing necroptosis in PMNs. The virulence factors released by MRSA after being phagocytosed by PMNs are recognized to promote the lysis of these neutrophils (Rungelrath et al., 2021). Specifically, USA300 has been shown to induce rapid lysis of these cells, resulting in the release of damage-associated molecular patterns (DAMPs), such as HMGB1 (Irene H et al., 2015; Ludwig et al., 2023). This lysis process appears to differ from apoptosis and is characterized by features of programmed necrosis or necroptosis (Greenlee-Wacker et al., 2017; Rungelrath et al., 2021; Song et al., 2021). During this process, severe inflammatory responses and the accumulation of reactive oxygen species (ROS) are often observed, exacerbating host tissue damage (Liu et al., 2023). Developing pharmacological agents that reduce virulence factor-induced PMN necroptosis is critical to mitigate immune-mediated inflammation in CA-MRSA infections.

Traditional Chinese medicine (TCM) has been widely studied for its antibacterial effects (Yang et al., 2018; Huang et al., 2020; Zhang et al., 2021; Zhang et al., 2022). Ginseng, as a well-known TCM with clear immunoregulatory properties, has also attracted attention for its antibacterial potential (Wang et al., 2020). Asian ginseng (*Panax ginseng* C.A. Meyer), American ginseng (*Panax quinquefolius*), and *Panax notoginseng* (PN) are the three primary ginseng species globally (Wang et al., 2020). Extracts from Asian ginseng and American ginseng have demonstrated significant inhibitory effects on the growth, adhesion, and pathogenicity of *Pseudomonas aeruginosa* (*P*. *aeruginosa*), *S. aureus* (*S*. *aureus*), and *Propionibacterium acnes* (Alipour et al., 2011; Wang et al., 2013; Zhang et al., 2014). However, most of these studies focus on the direct effects on the pathogens themselves, with little attention paid to the interaction between bacterial virulence factors and immune cell responses. Unlike the other two types of ginsengs, PN, derived from the dried roots and rhizomes of the Araliaceae family plant *Panax notoginseng* (Burk.) F.H. Chen, does not exhibit significant inhibition on bacterial growth. However, PN extracts significantly inhibit on the quorum sensing system, reducing the area of flagella motility-dependent swarming in *P*. *aeruginosa* (Koh and Tham, 2011). At the same time, PN, a common anti-inflammatory agent in TCM, possesses immunoregulatory and antioxidant effects (Nian-Wu et al., 2012; Liu et al., 2020; Sheng-Yan et al., 2024; Yang et al., 2024). Steamed PN (S-PN) exhibits strong pro-apoptotic effects on PMNs, promoting non-lytic apoptosis and thus clearing PMNs with minimal release of immune stimulants (such as histones and extracellular DNA) (Wang et al., 2019; Xiong et al., 2022; Yang et al., 2024). This suggests that S-PN may be an effective agent for modulating PMN immune responses and inhibiting inflammation, potentially aiding in the treatment of MRSA infections. Using an MRSA-PMN co-culture model, this study investigates the effects of S-PN on inflammatory responses and ROS accumulation in this model. RNA-seq analysis evaluates the impact of S-PN on the transcriptional expression of virulence factors in CA-MRSA USA300. Furthermore, the study explores the inhibitory effect of S-PN on the apoptosis of PMNs induced by CA-MRSA USA300. This study establishes a theoretical foundation for the clinical application of S-PN in the treatment of infectious diseases.

## 2 Materials and methods

### 2.1 Experimental reagents, bacterial strains and cells

S-PN was manufactured by Yunnan Qidan Pharmaceutical Co., Ltd., Yunnan, China. (Production Batch No: 2309003). Its preparation was performed according to the provisions of the Yunnan Province Food and Drug Administration, China (Yun YPBZ-0193-2013). The dried roots and rhizomes of PN (Araliaceae, *Panax notoginseng* (Burk.) F.H. Chen) were selected, cleaned, and steamed for 3 h. Afterward, they were dried and ground into a fine powder. The resulting powder is light yellow or brownish-yellow in color. The product quality standard stipulates that the content of total saponins of panax notoginseng (ginsenoside Rb1, ginsenoside Rg1, and ginsenoside R1) should not be less than 4.5% (Supplementary Figure S1; Supplementary Table S1). S-PN was dissolved in Dulbecco’s Modified Eagle Medium (DMEM) to prepare a 10 mg/mL S-PN solution, which was subsequently filtered through a 0.22 μm filter membrane to remove bacteria.

The MRSA standard strain USA300 (Kansas, United States), hereafter referred to as MRSA, was purchased from American type culture collection (ATCC). The strain was stored at −80°C. The strains were cultured on Mueller–Hinton (MH) agar broth (CM0337, Batch No. 2989738, OXOID, Britain) before use. MRSA passaging culture was performed using MH broth (CM0405, Batch No. 2963434, OXOID, Britain), which was incubated in THZ-D Desktop Constant Temperature Oscillator (Peiving Experimental Equipment Co., Ltd, Suzhou, China) at 37°C and 280 r/min with shaking until the log-phase growth was reached (16–18 h), after which the culture was diluted to 5 × 10^6^∼1 × 10^7^ CFU/mL using fresh medium.

The Ethics Committee of the Institute of Traditional Chinese Medicine Clinical and Basic Medical Sciences, China Academy of Chinese Medical Sciences approved all peripheral blood collection procedures. PMNs were isolated from the peripheral blood of healthy volunteers using a human peripheral blood neutrophil isolation solution kit (Batch No. P9402, Solarbio). The procedure involved adding 4 mL of neutrophil separation medium to a 10 mL sterile centrifuge tube, followed by carefully layering 10 mL of EDTA-anticoagulated peripheral blood along the wall of the tube to maintain separation. The sample was then centrifuged at 500 *g* for 30 min, resulting in six distinct layers, with neutrophils located in the fourth layer. The plasma, mononuclear cell layer, and separation medium were carefully removed, and the neutrophil layer was collected for subsequent use.

Trypsin-EDTA was purchased from Invitrogen (Carlsbad, California, United States). Tryptone (Batch No. LP0042) and yeast extracts (Batch No. LP0021) were obtained from Oxoid, United Kingdom. NaCl (Batch No. 10019318) was from Sinopharm Chemical Reagent Co., Ltd, China.

### 2.2 Cell viability assay

CCK-8 assay was recommended for viability test. In brief, PMNs (1 × 10^4^) were plated into a 96-well plate. Following 24 h of incubation, the medium or prepared S-PN (concentration range of 0.0312-10 mg/mL) was added to the wells and incubated for 24 h. The medium was then replaced with 100 μL serum-free medium, and 10 μL CCK-8 solution (Solarbio, China) was dropped into each well for 4 h of incubation. Absorbance was measured by microplate reader at 450 nm.

### 2.3 MRSA-PMN co-culture model

An *in vitro* model of MRSA-PMN infection was established based on methods reported in the literature (Chen et al., 2015; Singh et al., 2022), with modifications tailored to this study. PMNs were adjusted to a density of 1 × 10^4^ cells/well and uniformly seeded into plate wells. After 24 h of incubation at 37°C with 5% CO_2_, the supernatant was discarded. MRSA was cultured in MH medium to 10^8^–10^9^ CFU/mL, harvested by centrifugation at 3,000 rpm for 5 min, and washed three times with PBS. The bacterial pellet was resuspended in antibiotic-free DMEM. Revived MRSA was added to the wells at multiplicity of infection (MOI) ratios of 1:5, 1:10, and 1:20 (PMN:MRSA), followed by coculture at 37°C with 5% CO_2_ for 1, 4, and 18 h. The HMGB1 levels in the coculture supernatant were measured. MOI values and coculture durations that exhibited both elevated HMGB1 concentrations and statistically significant differences compared to other intervention conditions were selected as the optimal criteria for model establishment. Based on experimental results, an MOI of 1:10 and a 4 h coculture duration were selected as optimal parameters. For drug intervention, PMNs were pretreated with DMEM (MRSA-PMN group) or 10 mg/mL S-PN (S-PN-treated group) for 24 h prior to MRSA infection. The supernatant was then discarded, and MRSA-PMN coculture was performed as described above.

### 2.4 Enzyme-linked immunosorbent assay (ELISA)

ELISA was used to detect the secretion of HMGB1, IL-1β, IL-8, MCP-1, TNF-α in MRSA-PMN supernatants and PVL, PSM in MRSA. The sources of the kits were Human Interleukin 8 (IL-8) ELISA Kit (KQ110878), Human Tumor Necrosis Factor alpha (TNF-α) ELISA Kit (KQ102647), Human Interleukin 1β(IL-1β) ELISA Kit (KQ105768), Human Monocyte Chemotactic Protein 1 (MCP-1; CCL2) ELISA Kit (KQ110919), Human High Mobility Group Protein B1 (HMGB1) ELISA Kit (KQ105793), Bacterial phenol-soluble modulins (PSM) ELISA Kit (KQ141956) and Bacterial leucocytocide (PVL) ELISA kit (KQ141976) from Shanghai Keqiao Biotechnology Co., Ltd. Each sample was repeated three times.

### 2.5 Flow cytometry

Granulocyte counts were determined using BD FACSCalibur II™ Flow Cytometer. Granulocytes were identified and quantified by gating on the specific forward scatter (FSC) and side scatter (SSC) regions that correspond to their size and granularity. Intracellular ROS level was evaluated using the 2, 7-dichlorodihydrofluroresenin diacetate (DCFH-DA) (Cat.No. D399, Thermo). Cells were stained with 1 µL DCFH-DA for 60 min at 37°C, then washed and analyzed by flow cytometry. DCFH-DA fluorescence intensity (ex/em 488/525 nm) quantified intracellular ROS levels.

### 2.6 RNA extraction

Total RNA was extracted from three biological replicates of methicillin-resistant *S. aureus* (MRSA) in both control and S-PN-treated groups using TRIzol reagent (Invitrogen, CA, United States), followed by DNase I digestion (Takara, Japan) to eliminate genomic DNA. RNA integrity was rigorously verified through 1% agarose gel electrophoresis and Agilent 2,100 Bioanalyzer analysis (RIN ≥8.0; 28S/18S ratio >1.8), with quantification performed on a NanoDrop spectrophotometer (OD260/280 = 1.8–2.2).

### 2.7 RNA-seq

Sequencing libraries were constructed with NEBNext^®^ Ultra™ RNA Kit (NEB) following poly-A selection and cDNA synthesis protocols, and sequenced on Illumina NovaSeq 6,000 (150 bp paired-end). Raw reads were preprocessed through adapter trimming (Trimmomatic; LEADING/TRAILING:20) and quality filtering (Phred score ≥ Q20), then aligned to the *S. aureus* USA300_TCH1516 reference genome (NCBI Assembly GCA_000017885.1) using STAR (v2.7.9a) with ≤1 mismatch. SNP calling was performed using GATK3 HaplotypeCaller after duplicate removal (Picard v1.41) and base quality recalibration.

### 2.8 Bioinformatics analysis

Gene expression quantification using HTSeq (v0.5.4) generated FPKM-normalized counts through union counting mode with reverse strand specificity. Differentially expressed genes (DEGs) were identified using DESeq (v1.10.1) with a negative binomial model, requiring |log2 (fold change)| ≥1 and Benjamini–Hochberg adjusted P < 0.05. Functional enrichment of differentially expressed genes was assessed by GOseq (v1.34.0) with Wallenius distribution-based length bias correction and KOBAS 3.0 using the *S. aureus* USA300 KEGG pathway database (sau00001), applying hypergeometric testing with Benjamini-Yekutieli FDR correction (FDR < 0.1).

### 2.9 Quantitative real-time PCR (qRT‒PCR)

Total RNA was extracted using TRIzol reagent, and its quality was assessed with a NanoDrop spectrophotometer. cDNA was synthesized from 1 µg of RNA using HiScript III 1st Strand cDNA Synthesis Kit (+gDNA wiper) (R312-01, Nanjing Nuoweizan Biotechnology Co., Ltd). qRT-PCR was performed in a 20 µL reaction containing SYBR Green PCR Master Mix, primers (200 nM each), and cDNA template. The genes tested with MRSA as samples were *pvl*, *psmα1*, *psmα2*, *psmα3*, *psmα4* and the reference gene was 16s rRNA. The genes tested with PMNs as samples were ripk1, ripk3, mlkl, caspase-8, smac, spata2 and the reference gene was gapdh. The primer sequences used are shown in the Supplementary Table S2. The cycling conditions included an initial denaturation at 95°C for 10 min, followed by 40 cycles of 95°C for 15 s, annealing at the optimal temperature for 30 s, and extension at 72°C for 30 s. Gene expression was quantified using the ΔΔCt method, normalizing to reference gene.

### 2.10 Statistical analysis

Data are expressed as mean ± SD. Normality was assessed via Shapiro–Wilk test. For comparisons between two groups, unpaired Student’s t-test (parametric) or Mann–Whitney U test (non-parametric) was applied. One-way ANOVA with Tukey’s post-hoc test (parametric) or Kruskal–Wallis with Dunn’s test (non-parametric) was used for multi-group comparisons. Adjusted P values <0.05 were considered significant. Analyses were performed using GraphPad Prism v10.0.

## 3 Results

### 3.1 Effects of S-PN on PMNs in MRSA-PMN Co-culture

HMGB1 levels increased significantly in MRSA-exposed PMNs versus uninfected controls. At an MOI of 1:10 and an infection duration of 4 h, the levels of HMGB1 were elevated to a greater extent compared to MOIs of 1:5 or 1:20, or infection durations of 1 h or 18 h. Based on these data, an MOI of 1:10 and an infection duration of 4 h were selected as the conditions for establishing the MRSA-PMN model in subsequent experiments (Supplementary Figure S2A).

**FIGURE 1:**
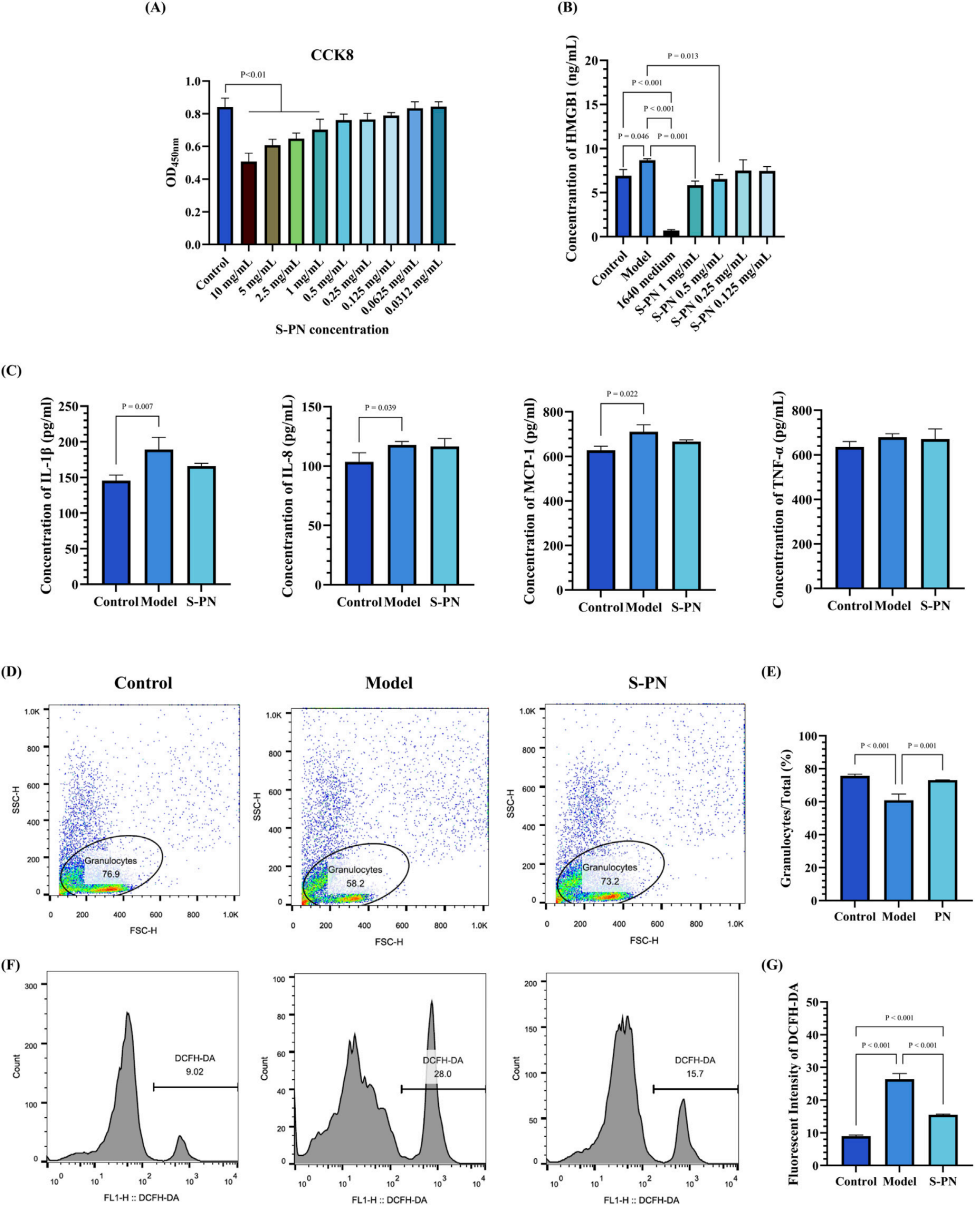
Effect of S-PN on PMNs. **(A)** Cytotoxicity of S-PN on PMNs. **(B)** Impact of S-PN on HMGB1 level released by PMNs in the MRSA-PMN model. **(C)** Expression levels of inflammatory cytokines in the MRSA-PMN model. **(D,E)** Flow cytometry analysis of the proportion of total PMNs in each group (n = 3). **(F,G)**. Flow cytometry analysis of ROS levels in each group (n = 3).

The MIC and MBC assays revealed that S-PN does not exhibit inhibitory effects on the growth of MRSA (Supplementary Figure S2B,C). In the evaluation of drug effects on the cell viability of PMNs, it was observed that S-PN exhibits cytotoxicity at concentrations exceeding 1 mg/mL, with a cell growth inhibition rate greater than 10% (Figure 1A). Treatment with 1 and 0.5 mg/mL S-PN resulted in a significant reduction in HMGB1 level in the MRSA-PMN system post-infection, compared to the untreated Model (MRSA-PMN) group (P < 0.05). These results suggest that S-PN protected PMNs from MRSA-induced damage (Figure 1B). In subsequent experiments of this study, 0.5 mg/mL S-PN, which exhibits no significant cytotoxicity to PMNs and effectively suppresses the MRSA-induced elevation of HMGB1 under PMN damage, will be selected as the optimal experimental drug concentration.

### 3.2 Inhibition of MRSA-Induced PMN inflammatory response by S-PN

S-PN was investigated for its inhibitory effects on MRSA-induced PMN inflammatory responses by assessing PMN inflammatory marker levels. Compared to the Control group, the levels of IL-1β, IL-8, and MCP-1 were significantly elevated in the Model group. In the S-PN group, IL-1β and MCP-1 showed a decreasing trend (Figure 1C). There were no significant differences in TNF-α levels between the Model and S-PN groups compared to the Control group. These results indicate that S-PN has a certain inhibitory effect on PMN inflammatory damage, reducing the release of MRSA-induced PMN pro-inflammatory factors.

### 3.3 Alleviation PMN apoptosis and ROS accumulation by S-PN

The cell numbers of PMNs and intracellular ROS levels were assessed to further determine the effects of MRSA on PMN-induced cell death and oxidative damage. The results showed that the number of granulocytes in the Model group was significantly reduced compared to the Control group, but this reduction was reversed following S-PN intervention (Figures 1D,E; Supplementary Figure S3). Analysis using DCFH-DA revealed a significant increase in ROS levels in the Model group compared to the Control group, while the S-PN group exhibited a notable decrease in ROS levels (Figures 1F,G; Supplementary Figure S3). These findings suggest that S-PN effectively reverses MRSA-induced oxidative damage in PMNs.

### 3.4 Inhibition of MRSA virulence factor-related pathways by S-PN

**FIGURE 2:**
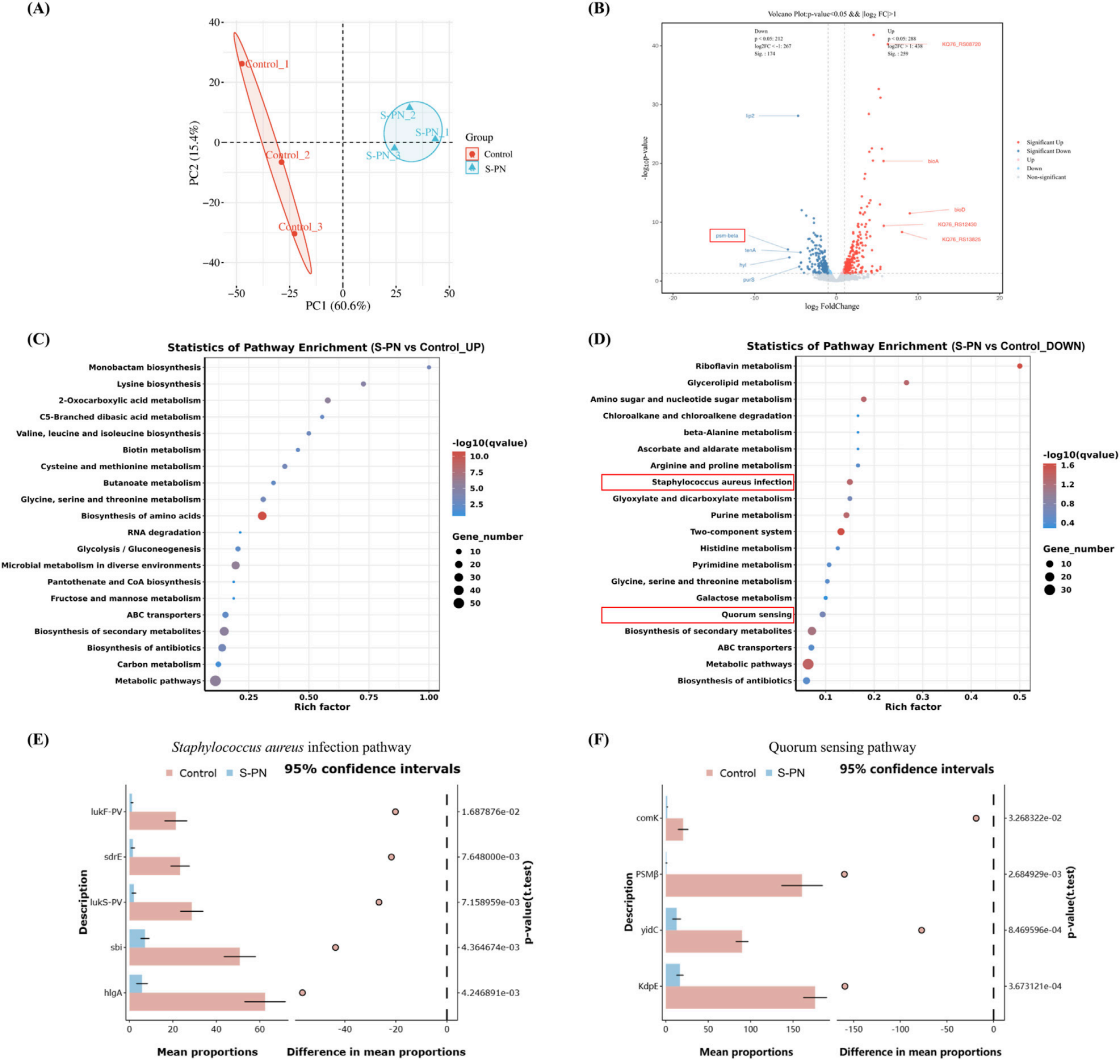
Effect of S-PN on MRSA Gene Transcription. **(A)** Principal Component Analysis (PCA) showing significant differences in gene transcription between MRSA with S-PN intervention (S-PN group) and MRSA without intervention (Control group). **(B)** The volcano plot. S-PN vs. Control, with red indicating significantly upregulated genes and blue indicating significantly downregulated genes (*P* < 0.05, |log_2_ FC| > 1). The top 10 genes with the most significant changes are labeled on the plot. **(C,D)**. KEGG pathway analysis showing significantly upregulated and downregulated pathways **(E,F)**. The difference expression levels of hub gene in each group in *Staphylococcus aureus* infection pathway and Quorum sensing pathway using STAMP.

To determine whether the anti-inflammatory effect of S-PN on MRSA-induced inflammation in PMNs is associated with transcriptional suppression of MRSA genes, RNA-seq was performed to analyze the transcriptome of MRSA USA300 strain before and after S-PN intervention. A total of 2033 genes were detected. Among these, 1826 genes were common to both the Control and S-PN groups, 180 genes were exclusively found in the Control group, and 27 genes were unique to the S-PN group (Supplementary Figures S4A,B). Significant transcriptional differences between the two groups were observed, with 276 DEGs identified (Figure 2A; Supplementary Table S3). Following S-PN intervention, 161 DEGs such as *uspA*, *bioA*, *bioD*, and *ribU* were significantly upregulated, while 115 DEGs including *psm-beta* (*psmβ*), *lip2*, *tenA*, *hyl*, and *purS* were significantly downregulated (Figure 2B; Supplementary Figure S4B).

Gene Ontology (GO) enrichment analysis revealed that the upregulated DEGs were primarily associated with biological processes (e.g., oxidation-reduction processes, carboxylic acid metabolic processes, and oxygen-containing compound metabolic processes), cellular components (e.g., ribosomes), and molecular functions (e.g., oxidoreductase activity and ribosome structural constituent). In contrast, the downregulated DEGs were predominantly linked to biological processes (e.g., lipid biosynthesis processes, vitamin metabolic processes, and water-soluble vitamin metabolic processes) and molecular functions (e.g., 3-beta-hydroxy-delta5-steroid dehydrogenase activity and steroid dehydrogenase activity with CH-OH group donors and NAD or NADP as acceptors) (Supplementary Figures S4C,D; Supplementary Table S4).

KEGG pathway enrichment analysis indicated that the upregulated DEGs were significantly enriched in amino acid metabolism pathways (e.g., lysine biosynthesis, cysteine and methionine metabolism, and valine, leucine, and isoleucine biosynthesis), carbon and sugar metabolism pathways (e.g., glycolysis/gluconeogenesis and butyrate metabolism), and other specific metabolism pathways (e.g., 2-oxoacid metabolism and C5-branched dibasic acid metabolism). Conversely, the downregulated DEGs were primarily enriched in vitamin metabolism pathways (e.g., riboflavin metabolism), carbon and sugar metabolism pathways (e.g., glycerolipid metabolism and amino sugar and nucleotide sugar metabolism), as well as pathogen infection and signaling pathways (e.g., *S. aureus* infection and quorum sensing) (Figures 2C,D; Supplementary Table S5).

The genes enriched in these two pathways of pathogen infection and signaling are *lukF-PV*, *lukS-PV*, *sdrE*, *sbi*, *hlgA*, and *clfB* (*S. aureus* infection), as well as *psmβ*, *ribD*, *sspA*, *comK*, *KdpE*, and *yidC* (*Quorum sensing*) (Supplementary Figure S5). Functionally, these genes are associated with pathogenicity-related processes such as cytolysis [*psmβ* (Ebner et al., 2017), *LukF-PV* (Okolie et al., 2013), *LukS-PV*(Okolie et al., 2013), *hlgA* (Venkatasubramaniam et al., 2018)], immune interference [*sbi* (Gonzalez et al., 2019), *sspA* (Oscarsson et al., 2006)], and bacterial adhesion and colonization [*sdrE* (Paiva et al., 2023), *clfB* (Xue et al., 2012)], as well as non-pathogenicity-related processes including bacterial genetic metabolism [*comK* (Turgay et al., 1997), *yidC* (Li et al., 2014), *ribD* (Miao et al., 2024)] and osmotic regulation [*KdpE* (Freeman et al., 2013)].

We observed that following S-PN intervention, the expression levels of cytolytic virulence factor genes, including *psmβ* (log2FC = −5.92), *LukF-PV* (log2FC = −2.28), *LukS-PV* (log2FC = −1.85), and *hlgA* (log2FC = −1.83), were significantly downregulated (Supplementary Figure S3E,F; Supplementary Figures S5A,C). *LukF-PV* and *LukS-PV* encode the CA-MRSA hallmark virulence factor PVL, while *psmβ* encodes PSM. PSM has been shown to enhance the lytic activity of PVL against PMNs (Hongo et al., 2009), whereas *hlgA* is primarily associated with erythrocyte lysis. Based on these findings, this study focuses on investigating the impact of S-PN on PVL- and PSM-mediated PMN apoptosis during CA-MRSA infection.

### 3.5 Inhibition of MRSA virulence factors PSM and PVL expression by S-PN

**FIGURE 3:**
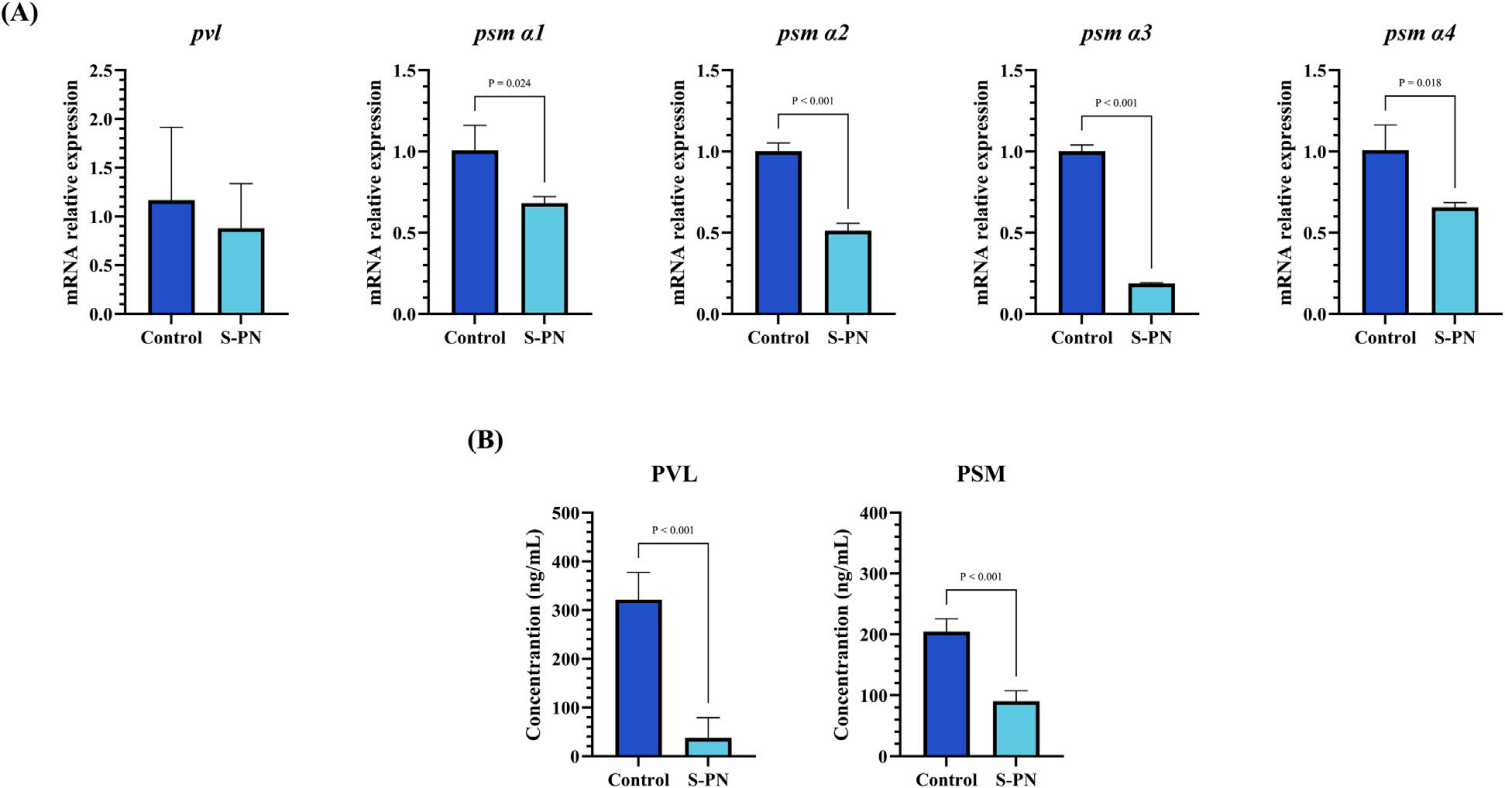
Effect of S-PN on MRSA virulence factors PSM and PVL expression. **(A)** The mRNA expression levels of MRSA virulence factors *psm α1, psm α2, psm α3, psm α4* and *pvl*. **(B)** The protein expression levels of MRSA virulence factors PSM and PVL.

The impact of S-PN on the expression of MRSA virulence factors PSM and PVL was assessed using PCR and ELISA. The mRNA transcription levels of *pvl*, *psm α1*, *α2*, *α3*, and *α4* in the S-PN group were significantly reduced compared to the Control group (Figure 3A). Similarly, the concentration levels of PVL and PSM in cells were markedly decreased in the S-PN group compared to the Control group (Figure 3B). These results indicate that S-PN exerts a suppressive effect on both the transcription and expression of MRSA virulence factors PSM and PVL. Therefore, S-PN may reduce the pathogenicity of MRSA by modulating its virulence factors.

### 3.6 Inhibitory effects of S-PN on the RIPK1/RIPK3/MLKL pathway induced by MRSA in PMNs

**FIGURE 4:**
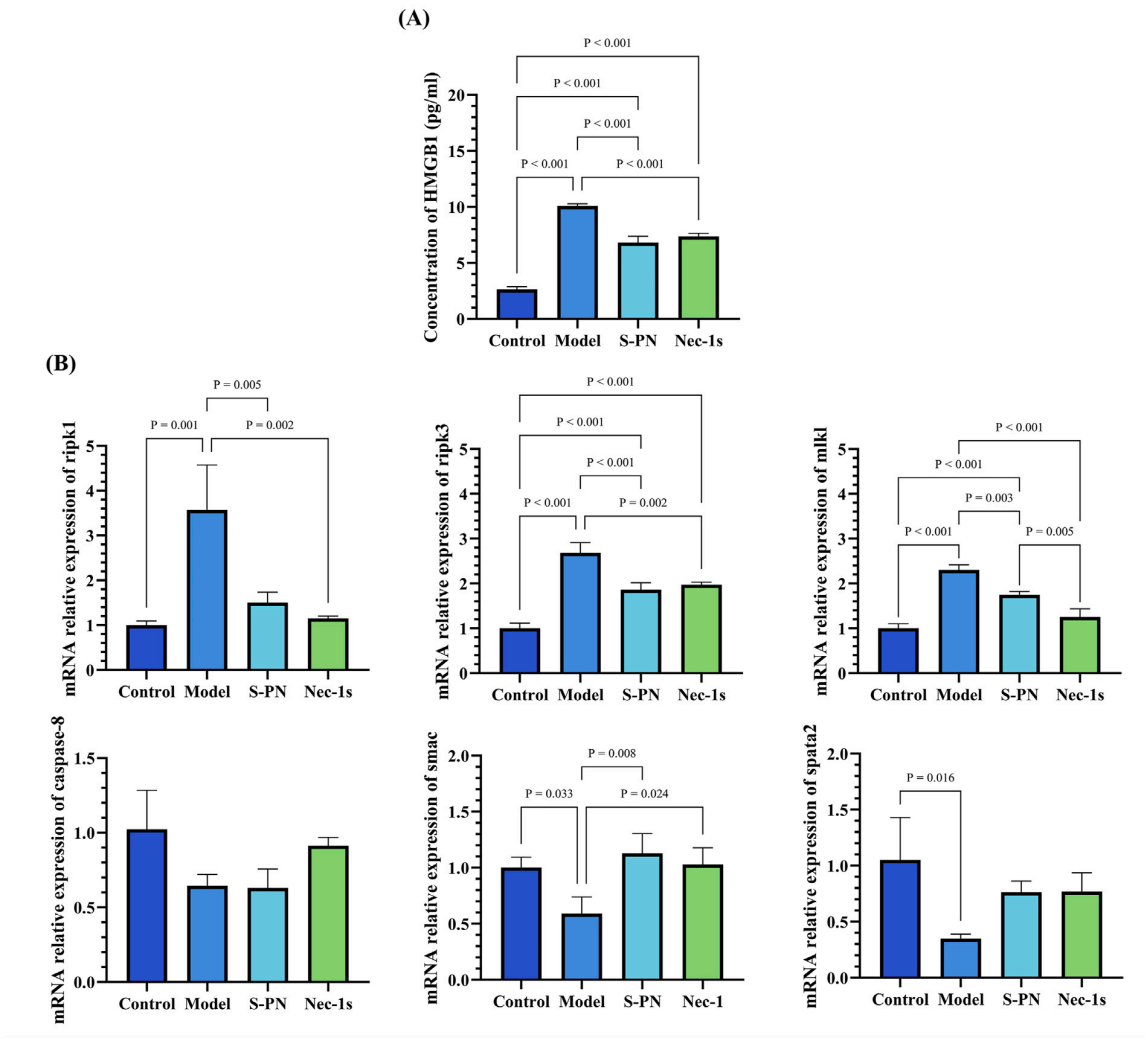
Effects of S-PN on the Necroptotic Apoptosis Pathway in MRSA-PMNs. **(A)** Expression levels of HMGB1 in MRSA-PMNs. **(B)** Expression levels of genes associated with the necroptotic apoptosis pathway in PMNs.

To further explore the specific mechanisms by which MRSA virulence factors induce damage in PMNs, we examined the expression levels of genes associated with RIPK1/RIPK3/MLKL necroptotic signaling pathway in PSM-induced PMNs. Necrostatin-1s (Nec-1s), an RIPK1 inhibitor, was used as a control (Supplementary Figure S2D). Both the S-PN and Nec-1s groups exhibited similar inhibitory effects on HMGB1 compared to the Control group, indicating that suppression of the necroptotic pathway can mitigate PMN damage (Figure 4A). In terms of gene expression related to the necroptotic pathway, the mRNA levels of ripk1, ripk3, and mlkl were significantly elevated in the model group, suggesting that MRSA induces necroptosis in PMNs. Following S-PN intervention, the mRNA levels of ripk1, ripk3, and mlkl were significantly reduced, with ripk1 and ripk3 levels comparable to those in Nec-1s group (Figure 4B). These results suggest that S-PN may regulate PMN necroptosis by inhibiting the transcription of ripk1, ripk3, and mlkl mRNA.

This finding was further corroborated by analyzing the mRNA expression of relevant regulators of the RIPK1/RIPK3/MLKL pathway, including caspase-8, smac, and spata2 (Figure 4B). Compared to the Control group, the expression of smac and spata2 was significantly decreased in the Model group, while caspase-8 expression also showed a decreasing trend (Figure 4B). The decreased expression of caspase-8 suggests that MRSA may promote necroptotic signaling by reducing the ability of Caspase-8 to dissociate the RIPK1/RIPK3 complex, thereby maintaining the complex and facilitating necroptosis. The decreased spata2 expression in the Model group suggests that necroptotic signaling in PMNs is not activated by MRSA through the enhancement of SPATA2-mediated RIPK1 deubiquitination. The S-PN group showed a trend of increased smac expression and similar levels of caspase-8 expression compared to the Model group, suggesting that S-PN may enhance SMAC-mediated activation of Caspase, though this activation does not appear to target caspase-8. The exact mechanism warrants further investigation.

## 4 Discussion

CA-MRSA spreads rapidly and has increasingly become predominant in community infections across various regions worldwide (Tenover and Goering, 2009). Its pathogenicity largely depends on its virulence factors. CA-MRSA virulence factors directly suppress host immune responses. As the first line of innate immunity, PMNs are particularly vulnerable to MRSA virulence factors like PSM, which can inhibit their phagocytic and bactericidal activities while inducing necroptosis (Peschel and Otto, 2013). S-PN, a commonly used anti-inflammatory agent in TCM, exhibits clear necroptosis inhibition (Hu et al., 2022). In this study, using an MRSA-PMNs co-culture model, we observed that S-PN suppressed the inflammatory response and ROS accumulation in the model. Furthermore, S-PN significantly inhibits the transcription of CA-MRSA USA300 virulence factors, including PSM and PVL, and reduces the expression of pathogen infection-related pathways, including quorum sensing. This leads to the suppression of necroptotic signaling pathways induced by PSM in PMNs.

**FIGURE 5:**
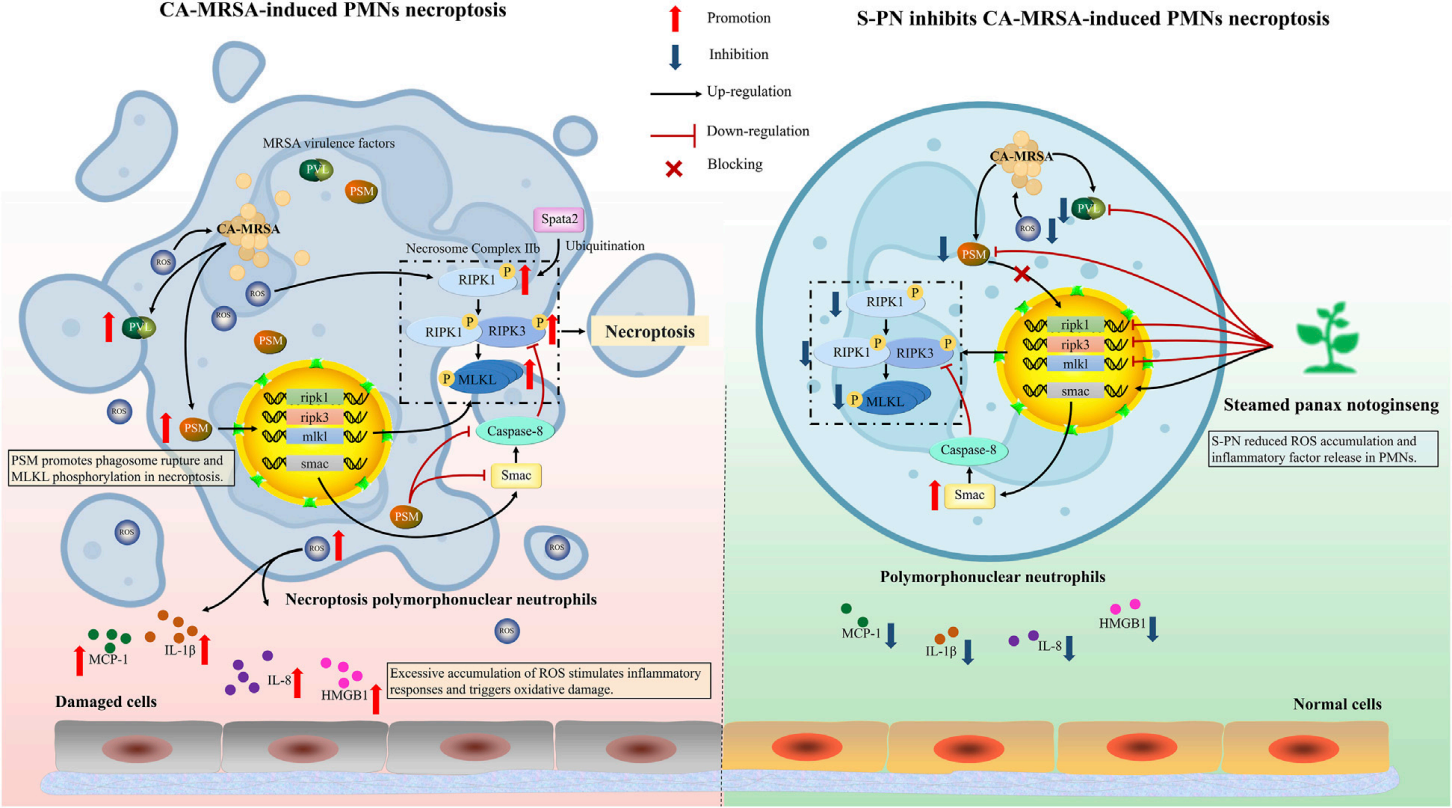
Schematic of the mechanism of S-PN inhibits CA-MRSA-induced necroptosis in PMNs. *(Left panel)* Upon MRSA phagocytosis by PMNs, robust ROS production facilitates bacterial clearance; however, MRSA counteracts this defense through secretion of virulence factors PVL and PSMs, which activate PSM-mediated necroptosis pathways in PMNs, ultimately triggering neutrophil lysis and exacerbating inflammatory responses. *(Right panel)* S-PN intervention suppresses MRSA virulence factor release, thereby abrogating PMN necroptosis and mitigating pathological inflammation. PVL, Panton-Valentine Leukocidin; PSM, Phenol Soluble Modulin; ROS, Reactive Oxygen Species; RIPK1, Receptor-Interacting Protein Kinase 1; RIPK3, Receptor-Interacting Protein Kinase 3; MLKL, Mixed-Lineage Kinase Domain-Like; SMAC, Second Mitochondria-Derived Activator of Caspases; SPATA2, Spermatogenesis Associated 2; IL-8, Interleukin-8; IL-1β, Interleukin-1 Beta; HMGB1, High Mobility Group Box 1; MCP-1, Monocyte Chemoattractant Protein-1. Image created by Figdraw.

PMNs are the most abundant white blood cells in the circulatory system, and their phagocytic activity is crucial for the clearance of invading MRSA. The surface Toll-like receptors (TLRs) on PMNs recognize various surface-associated or freely secreted molecules produced by MRSA, thereby activating phagocytosis of MRSA (Hayashi et al., 2003). Intracellular ROS disrupt bacterial redox homeostasis, inducing lethal oxidative stress (Valko et al., 2007). However, excessive ROS can also be released into the extracellular environment, leading to localized tissue damage at the infection site (Figure 5). Research has demonstrated that ROS can induce permanent changes in the expression of inflammatory genes through redox signaling (Wright et al., 2010). Additionally, ROS can activate the NLRP3 inflammasome, leading to the cleavage of inflammatory cytokine precursors pro-IL-1β and pro-IL-18 into their active forms (IL-1β, IL-18) (Zhou et al., 2010). To prevent severe inflammatory responses and damage to host cells and tissues caused by excessive ROS accumulation, regulating the balance of ROS within PMNs is crucial for effective anti-infection therapy (Figure 5). S-PN demonstrates potent free radical scavenging (Xiong et al., 2019). Our findings confirm its antioxidative efficacy in combating CA-MRSA infection, with significantly reduced ROS accumulation levels observed in MRSA-PMN following S-PN intervention.

Current studies have confirmed that reducing excessive ROS production in glioblastoma cells can decrease pro-inflammatory factors IL-8 and MCP-1, thereby preventing the establishment of a harmful pro-inflammatory tumor microenvironment (Tewari et al., 2009). MCP-1 regulates immune cell recruitment (PMNs, monocytes, lymphocytes) to damaged tissues (Deshmane et al., 2009). It is primarily produced by early-infiltrating PMNs during infection. Inhibition of this chemokine can block the recruitment of immune cells to the infection site, thereby reducing excessive inflammatory responses (Yoshimura and Takahashi, 2007; Deshmane et al., 2009). In this study, MRSA caused significant damage to PMNs, with notable increases in the pro-inflammatory factors IL-1β, IL-8, and MCP-1. The anti-inflammatory efficacy of PN has been well-established in scientific literature. However, our results demonstrated only modest downregulation of inflammatory mediators following S-PN intervention. This attenuated effect may be attributed to alterations in bioactive components during PN processing (Xiong et al., 2019). While ginsenoside 20(S)-Rg3 has been identified as the primary bioactive component responsible for S-PN’s anti-inflammatory effects, its content in S-PN is comparatively lower than unprocessed PN-derived anti-inflammatory constituents such as notoginsenoside R1, ginsenosides Rg1, Re, and Rb1 (Zhang et al., 2019). Although the intervention with S-PN did not significantly downregulate inflammatory factors. Reduced HMGB1, elevated granulocytes, and suppressed ROS collectively indicate that S-PN provides protective effects for PMNs (Figure 5).

Current research on the regulation of PMN immune function by PN primarily focuses on pathological conditions such as myocardial infarction (Li et al., 2022), ischemia-reperfusion (Rong et al., 2009), and inflammation (Xiong et al., 2022). However, the regulatory effect of S-PN on PMNs during infectious diseases, where external pathogens invade the body, has not been thoroughly explored. In the process of CA-MRSA infection, its strategy to counteract host immune defense relies on its functional agr quorum sensing system (Cheung et al., 2021; Cella et al., 2023). The high virulence phenotype exhibited by CA-MRSA USA300 is intricately linked to the activation of the agr system. The quorum sensing system regulates the production of virulence factors that can evade PMN-mediated killing and lead to rapid destruction of these host cells (Matsumoto et al., 2021). Our study demonstrates that S-PN significantly downregulates the quorum sensing pathway in the MRSA-PMN model, suggesting that S-PN may influence the production of virulence factors regulated by this pathway. Consequently, we further investigated the virulence factors of USA300. Traditional programmed cell death is characterized by non-lytic processes. During cell death, ‘eat me’ signals attract phagocytes to clear the dead cells without triggering an inflammatory response (Newton et al., 2024). In contrast, lytic apoptosis, including necroptosis, involves cell rupture during death, releasing cellular contents and triggering an inflammatory response (Newton et al., 2024). PVL, a two-component toxin composed of LukF-PV and LukS-PV, has been shown to induce PMN lysis, impair host defenses, and facilitate the clearance of infected tissues (Jhelum et al., 2024). PVL-containing CA-MRSA strains typically cause tissue necrosis and leukopenia (Hofstee et al., 2024). PVL can activate the mitochondrial pathway of caspases to induce spontaneous apoptosis in PMNs (Genestier et al., 2005). However, there have been no clear reports indicating that PVL is associated with the necroptotic pathway in PMNs.

Another critical virulence factor of USA300, PSM, is an amphipathic α-helical peptide with strong hemolytic activity, considered a key factor in enhancing CA-MRSA virulence (Kim et al., 2020). Different types of PSM exhibit varied effects on PMNs (Hu Z. et al., 2022). PSMα3, in particular, has notable pro-inflammatory activity, stimulating PMN chemotaxis and inducing IL-8 release (Matsumoto et al., 2021). PSMα1, PSMα2, and PSMα3 contribute to the enhanced cell-lytic activity of CA-MRSA. Compared to PSMα gene knockout mutants, wild-type strains exhibit significantly higher lytic activity against PMNs and monocytes (Wang et al., 2007). Research reports indicate that after phagocytosing *S. aureus*, PMNs undergo dissolution, accompanied by characteristics of both apoptosis and necrosis (Chen H. et al., 2022). This is due to PSMα1, α2, and α3 inducing MLKL phosphorylation, leading to necroptotic apoptosis in PMNs (Zhou et al., 2018). The necroptosis pathway primarily involves the RIPK1 and RIPK3 proteins, which interact to activate the apoptotic program (Figure 5). RIPK complex-mediated MLKL phosphorylation drives its oligomerization and membrane translocation, where it interacts with phosphatidylinositol, triggering membrane permeabilization and cell destruction (Rickard et al., 2014; Wang et al., 2014; Chen et al., 2017). S-PN suppresses the formation of lytic programmed cell death mechanisms, such as neutrophil extracellular traps (NETosis) (Chen Z. et al., 2022). In this study, experiments were conducted to investigate whether S-PN blocks CA-MRSA-induced necroptosis, a similarly lytic form of cell death. The results demonstrated that S-PN suppresses the expression of PVL and PSM. This indicates that S-PN interferes with the induction and activation of the necroptosis pathway in PMNs by PSM through downregulating the quorum sensing pathway in USA300. Specifically, this activation is confirmed to enhance the transcription of key genes ripk1, ripk3, and mlkl, which is reversed by S-PN (Figure 5). The inhibitory effects on ripk1 and ripk3 align with those of the RIPK1 inhibitor Nec-1s, while the suppression of mlkl is slightly weaker compared to Nec-1s. Nec-1s directly binds to the RIPK1 protein and inhibits its phosphorylation, thereby reducing MLKL activation (Jhun et al., 2019). Regarding the impact on necroptosis pathway-related proteins, based on existing studies, we hypothesize that PSM induces MLKL phosphorylation, promotes MLKL interaction with cell membranes, and triggers cell lysis. By suppressing PSM expression in USA300, S-PN reduces PSM-mediated MLKL phosphorylation and blocks PMN lysis. Notably, S-PN not only exhibits anti-necroptotic effects comparable to Nec-1s but also directly targets the pathogen by inhibiting the quorum sensing pathway and virulence factor expression in CA-MRSA.

Necroptosis is regulated by multiple intracellular factors. The inhibition of necroptosis is controlled by Caspase-8, which mediates the classical apoptotic pathway by cleaving RIPK1 protein to suppress its kinase activity, thereby blocking necroptosis (Weinlich et al., 2017). Smac, a caspase activator, facilitates the activation of downstream caspases and assists the Caspase-8 complex in inducing apoptosis and suppressing necrosis (Figure 5) (He et al., 2009; Green, 2022). Studies have shown that S-PN is enriched with bioactive components such as ginsenoside Rh4 and 20(S)-Rg3, which induce apoptosis in PMNs at amputation sites in zebrafish larvae (Xiong et al., 2022; Yang et al., 2024). However, our experimental results revealed that although S-PN significantly enhances smac transcription, it does not increase caspase-8 expression, indicating that S-PN does not inhibit the necroptosis pathway via Caspase-8-mediated RIPK1 cleavage. Further studies demonstrate that active components in S-PN, including ginsenoside F4 and ginsenoside Rg6, exert significant regulatory effects on the mitochondria-dependent apoptotic protein Bax and the anti-apoptotic protein Bcl-2 (Chen and Jia, 2013; Chen et al., 2013). We hypothesize that S-PN may activate the classical apoptotic pathway by upregulating Bax and suppressing Bcl-2, thereby inhibiting necroptosis. Additionally, Spata2, a deubiquitinating enzyme for M1 ubiquitin chains of RIPK1, promotes necroptosis by enhancing RIPK1 kinase activity (Wei et al., 2017). MRSA suppresses spata2 transcription, suggesting that its promotion of PMN necroptosis does not rely on modulating RIPK1 ubiquitination. Nevertheless, whether MRSA or its virulence factor PSM directly regulates RIPK1 requires further investigation.

## 5 Conclusion

The induction of PMN necroptosis by CA-MRSA virulence factors is one of the key challenges in treating MRSA infections. During CA-MRSA infection, severe inflammatory responses caused by ROS accumulation in PMNs also contribute to tissue damage. S-PN reduces ROS levels in PMNs, inhibits the release of inflammatory cytokines IL-1β and IL-8, and suppresses the production of the chemokine MCP-1. By downregulating the quorum sensing system, S-PN inhibits the production of CA-MRSA virulence factors PVL and PSM, thereby blocking PSM-induced necroptosis in PMNs. This results in reduced expression of ripk1, ripk3, and mlkl, ultimately diminishing CA-MRSA’s pro-necroptotic effects on PMNs. This study demonstrates that S-PN protects host immune defenses by suppressing CA-MRSA virulence factor release, thereby preserving PMN-mediated immune functions and mitigating tissue injury caused by excessive inflammation. Future research will focus on validating S-PN’s therapeutic efficacy against CA-MRSA infections in in vivo models. Our findings provide a theoretical foundation for the clinical application of traditional Chinese medicine in antibacterial therapy.

## Data Availability

The data presented in the study are deposited in the Gene Expression Omnibus (GEO) database, accession number GSE284429.
