# Supplementary material for: Steamed panax notoginseng mitigates CA-MRSA USA300-induced necroptosis in human neutrophils
Source: Front Pharmacol. 2025 May 30;16:1546652. doi: 10.3389/fphar.2025.1546652 (PMC12163054; doi:10.3389/fphar.2025.1546652)
Supplement: Supplementary file 1 [file Supplementaryfile1.docx]

Supplementary Material

# Quality control verification of steamed panax notoginseng (S-PN)

The quality control of the S-PN powder used in this study was conducted according to the standards of the Yunnan Province Food and Drug Administration, China (Yun YPBZ-0193-2013). Weigh 0.5 g of S-PN powder, add 5 drops of water, and mix thoroughly. Then, add 5 mL of water-saturated butanol, shake for 10 min, and allow the mixture to stand for 2 h. Centrifuge and collect the supernatant. Add 3 times the volume of water-saturated butanol to the supernatant, shake well, and let it stand to allow phase separation. Collect the butanol layer and evaporate to dryness. Dissolve the residue in 1 mL of methanol to prepare the test solution. Ginsenoside Rb1, ginsenoside Rg1, and ginsenoside R1 were used to prepare a mixed solution of total saponins from panax notoginseng as the reference standard. Both the reference standard solution (panax notoginseng saponins) and the test sample (S20230401-1, S20231113-1, S20240201-1) solution were injected in a volume of 10 µL. The chromatographic separation was performed using an octadecylsilane-bonded silica column with acetonitrile as mobile phase A and water as mobile phase B. A gradient elution was employed as follows: 0-12 min with mobile phase A at 19% and mobile phase B at 81%; 12-60 min with mobile phase A at 36% and mobile phase B at 64%. The detection wavelength was set to 203 nm. The theoretical plate count for ginsenoside R1 peak should not be less than 4000. The total saponins of panax notoginseng should not be less than 4.5%. The detection results of the quality control are shown in Figure S1 and Table S1.


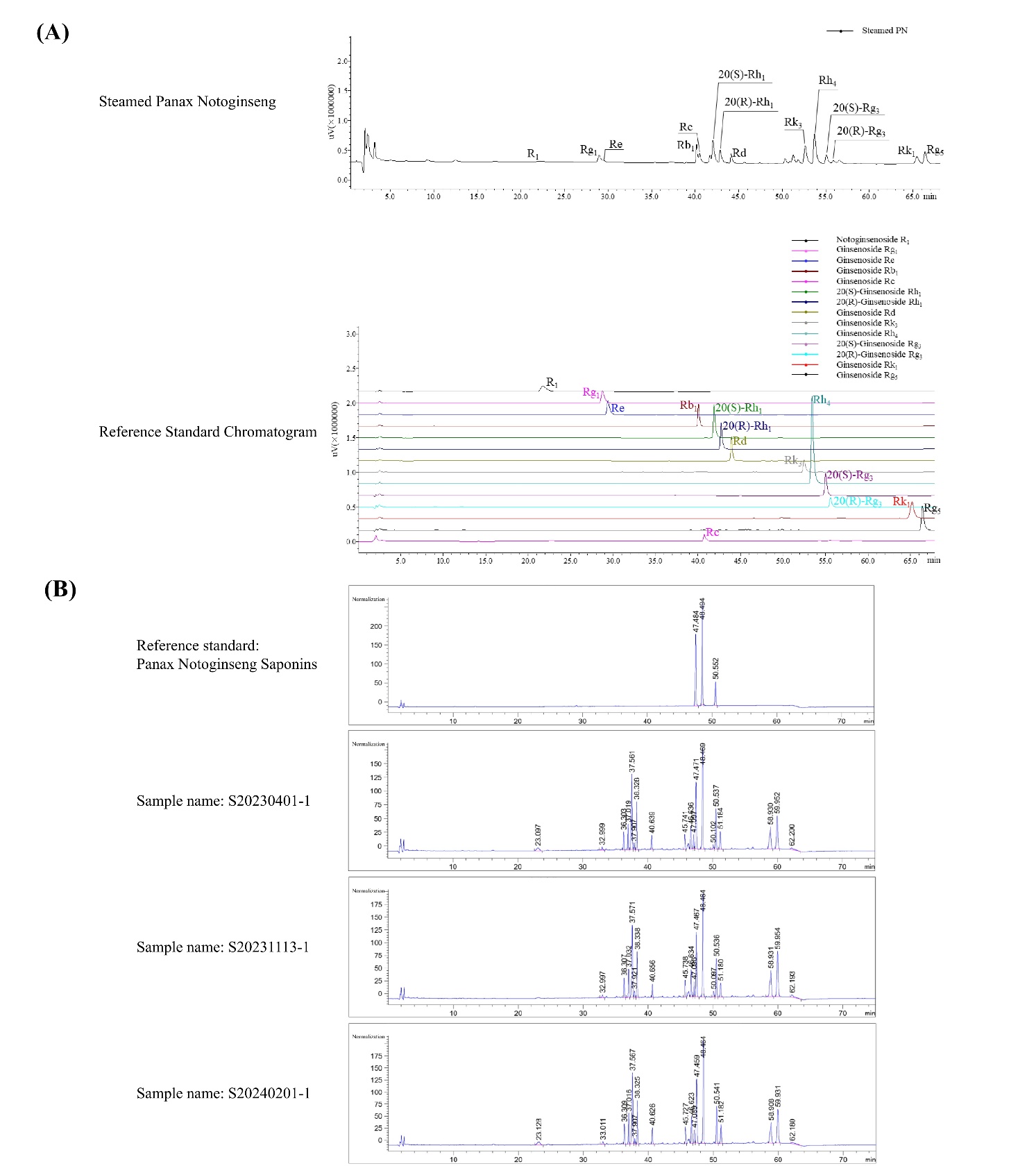


**Supplementary Figure S1. Characteristic chromatogram and quantitative chromatogram of S-PN. (**A). Characteristic chromatogram of S-PN; (B). Quantitative chromatogram of S-PN.

**Table S1. HPLC-MS analysis parameters and performance indexes of S-PN**

| **Sample name** | **Retention time (min)** | **Peak area (mAU*s)** | **Peak Height** | **Symmetry Factor** | **Peak Width** | **Number of Theoretical Plates** | **Resolution** | **Selectivity** |
| --- | --- | --- | --- | --- | --- | --- | --- | --- |
| Reference standard | 47.484 | 1518.3593 | 171.10049 | 0.98 | 0.1383 | 652780 | - | - |
|  | 48.494 | 2177.5872 | 244.42104 | 1.04 | 0.1417 | 649182 | 4.24 | 1.02 |
|  | 50.552 | 519.99219 | 57.06786 | 1.06 | 0.1433 | 689103 | 8.49 | 1.04 |
| S20230401-1 | 23.097 | 134.25928 | 4.54591 | 0.97 | 0.47 | 13379 | - | - |
|  | 32.999 | 52.40396 | 5.01639 | 0.99 | 0.1217 | 407498 | 19.66 | 1.43 |
|  | 36.303 | 218.90903 | 29.05427 | 0.96 | 0.1178 | 526373 | 16.21 | 1.1 |
|  | 37.019 | 270.46494 | 42.03888 | 1.1 | 0.1008 | 746643 | 3.85 | 1.02 |
|  | 37.561 | 1128.717 | 123.44836 | 1.32 | 0.1356 | 425346 | 2.7 | 1.01 |
|  | 37.907 | 78.33608 | 10.65854 | 0.95 | 0.1173 | 578249 | 1.61 | 1.01 |
|  | 38.328 | 622.89148 | 79.29752 | 0.99 | 0.1222 | 544788 | 2.06 | 1.01 |
|  | 40.639 | 155.95679 | 23.1044 | 0.99 | 0.1067 | 804088 | 11.86 | 1.06 |
|  | 45.741 | 197.79221 | 24.117 | 0.99 | 0.1283 | 703780 | 25.51 | 1.13 |
|  | 46.636 | 309.8028 | 37.49769 | 0.97 | 0.13 | 713003 | 4.07 | 1.02 |
|  | 47.097 | 190.35168 | 23.32252 | 0.98 | 0.13 | 727138 | 2.08 | 1.01 |
|  | 47.471 | 986.09589 | 109.85942 | 0.96 | 0.1411 | 626959 | 1.62 | 1.01 |
|  | 48.469 | 1581.4753 | 166.58859 | 1 | 0.1467 | 605070 | 4.08 | 1.02 |
|  | 50.102 | 76.88586 | 7.14788 | 1.01 | 0.1667 | 500611 | 6.12 | 1.03 |
|  | 50.537 | 608.80115 | 66.13294 | 1.09 | 0.1456 | 667848 | 1.64 | 1.01 |
|  | 51.184 | 283.18832 | 29.53581 | 0.99 | 0.15 | 645055 | 2.57 | 1.01 |
|  | 58.93 | 656.27795 | 36.19173 | 1.72 | 0.3067 | 204579 | 19.93 | 1.15 |
| S20231113-1 | 32.997 | 57.54678 | 5.25452 | 0.98 | 0.125 | 386006 | - | - |
|  | 36.307 | 261.23645 | 34.63485 | 0.95 | 0.1189 | 516615 | 15.95 | 1.1 |
|  | 37.032 | 327.88348 | 49.98354 | 1.12 | 0.1022 | 727064 | 3.86 | 1.02 |
|  | 37.571 | 1201.6277 | 127.79529 | 1.35 | 0.1389 | 405418 | 2.63 | 1.01 |
|  | 37.921 | 84.07696 | 11.25789 | 0.95 | 0.1183 | 568954 | 1.6 | 1.01 |
|  | 38.338 | 644.30286 | 81.214 | 0.97 | 0.1244 | 525814 | 2.02 | 1.01 |
|  | 40.656 | 158.62502 | 23.67487 | 1.01 | 0.1056 | 821830 | 11.84 | 1.06 |
|  | 45.738 | 247.70865 | 29.76419 | 1 | 0.13 | 685787 | 25.35 | 1.12 |
|  | 46.634 | 397.86969 | 47.7632 | 0.98 | 0.1322 | 689176 | 4.02 | 1.02 |
|  | 47.096 | 220.32004 | 27.281 | 0.99 | 0.1283 | 746081 | 2.08 | 1.01 |
|  | 47.467 | 1015.0609 | 113.93211 | 0.95 | 0.1389 | 647034 | 1.63 | 1.01 |
|  | 48.464 | 1675.5892 | 181.57494 | 1.01 | 0.1433 | 633399 | 4.15 | 1.02 |
|  | 50.097 | 109.06503 | 10.40918 | 1.01 | 0.1622 | 528303 | 6.28 | 1.03 |
|  | 50.536 | 633.24969 | 67.68469 | 1.12 | 0.1478 | 647860 | 1.66 | 1.01 |
|  | 51.18 | 237.81311 | 24.79895 | 0.97 | 0.1483 | 659492 | 2.56 | 1.01 |
|  | 58.931 | 872.59143 | 46.38624 | 1.86 | 0.32 | 187893 | 19.45 | 1.15 |
|  | 59.954 | 1126.0747 | 80.3812 | 1.06 | 0.22 | 411452 | 2.23 | 1.02 |
| S20240201-1 | 23.128 | 168.51028 | 5.59147 | 1.04 | 0.4833 | 12685 | - | - |
|  | 33.011 | 53.00198 | 5.4026 | 0.91 | 0.1183 | 431136 | 19.3 | 1.43 |
|  | 36.309 | 282.42383 | 37.8883 | 0.98 | 0.1178 | 526478 | 16.41 | 1.1 |
|  | 37.016 | 370.45193 | 58.11869 | 1.09 | 0.0992 | 771964 | 3.83 | 1.02 |
|  | 37.567 | 1261.1277 | 133.65123 | 1.37 | 0.14 | 398917 | 2.7 | 1.01 |
|  | 37.907 | 75.90599 | 10.45417 | 0.94 | 0.116 | 591628 | 1.56 | 1.01 |
|  | 38.325 | 646.21429 | 82.63904 | 1 | 0.1233 | 534932 | 2.05 | 1.01 |
|  | 40.626 | 213.90201 | 31.19005 | 0.99 | 0.1078 | 787090 | 11.7 | 1.06 |
|  | 45.727 | 259.48679 | 31.74674 | 0.98 | 0.1289 | 697329 | 25.33 | 1.13 |
|  | 46.623 | 426.311 | 50.71627 | 0.97 | 0.1333 | 677323 | 4.01 | 1.02 |
|  | 47.089 | 207.65543 | 25.2872 | 1 | 0.1308 | 717711 | 2.08 | 1.01 |
|  | 47.459 | 1059.8688 | 120.57716 | 0.96 | 0.1378 | 657302 | 1.62 | 1.01 |
|  | 48.464 | 1793.5866 | 193.27658 | 1.02 | 0.1433 | 633374 | 4.2 | 1.02 |
|  | 50.541 | 643.2027 | 70.01582 | 1.11 | 0.1444 | 678260 | 8.48 | 1.04 |
|  | 51.182 | 336.56189 | 35.69678 | 1.04 | 0.1483 | 659554 | 2.58 | 1.01 |
|  | 58.908 | 743.40417 | 39.23622 | 1.84 | 0.3283 | 178330 | 19.04 | 1.15 |
|  | 59.931 | 898.93878 | 63.90266 | 1.03 | 0.2217 | 404960 | 2.19 | 1.02 |

# Effect of different multiplicities of infection (MOI) on HMGB1 levels in MRSA-infected PMNs

Before establishing the MRSA-PMNs model, different multiplicities of infection (MOI) ratios (PMN:MRSA) were set at 1:5, 1:10, and 1:20, with infection durations of 1 h, 4 h, and 18 h. The level of HMGB1 in the cell supernatant was measured to assess the extent of bacterial infection. The results indicated that an MOI of 1:10 and an infection time of 4 hours resulted in the most severe infection of PMNs by MRSA (Figure S2A).

# Minimum inhibitory concentration (MIC) and minimal bactericidal concentration (MBC) of S-PN against MRSA

The antibacterial activity of S-PN against MRSA was determined using the broth microdilution method (Fankam et al., 2011). Two-fold serial dilutions were made in Luria-Bertani (LB) broth over a range to give final concentrations of 0.0312-40 mg/mL for S-PN solution and 0.0625–32 μg/mL for vancomycin. Then 100 μL of bacterial suspension (5×106 ~1×107 CFU/mL) was added to each well. The negative control was comprised of LB broth and the tested sample while the positive control was LB broth and bacterial suspension only. The final volume of each well was 200μL. The MICs of the test samples were detected after 24 h of incubation at 37 °C, followed by the addition of 30 μL of 2.5 mg/mL TTC and incubation for an additional 20 min at 37 °C (Yang et al., 2018). Viable bacteria reduced the yellow dye to pink. The inoculating loop was used to pick up the liquid from the corresponding wells of the 96-well plate and then inoculate it onto LB agar plates. After incubating at 37°C for 24 h, bacterial growth was observed, and the lowest concentration at which no visible bacterial colonies appeared was considered MBC of the drug.

The result is shown in Figure S2B and S2C. In comparison to the positive control vancomycin, which exhibited a MIC of 2 µg/ml against MRSA. But S-PN did not inhibit the growth of MRSA within the concentration range of 0.0312-40 mg/mL (Figure S2C).

# The impact of RIPK1 inhibitor on PMNs growth

In this study, the RIPK1 inhibitor Necrostatin-1 (Nec-1s) was used as a positive control reagent to investigate the effects of S-PN on necroptotic apoptosis pathway genes in PMNs. According to the CCK-8 assay, Nec-1s did not exhibit any cytotoxic effects on PMNs within the concentration range of 1.56 to 200 µg/mL (Figure S2D).


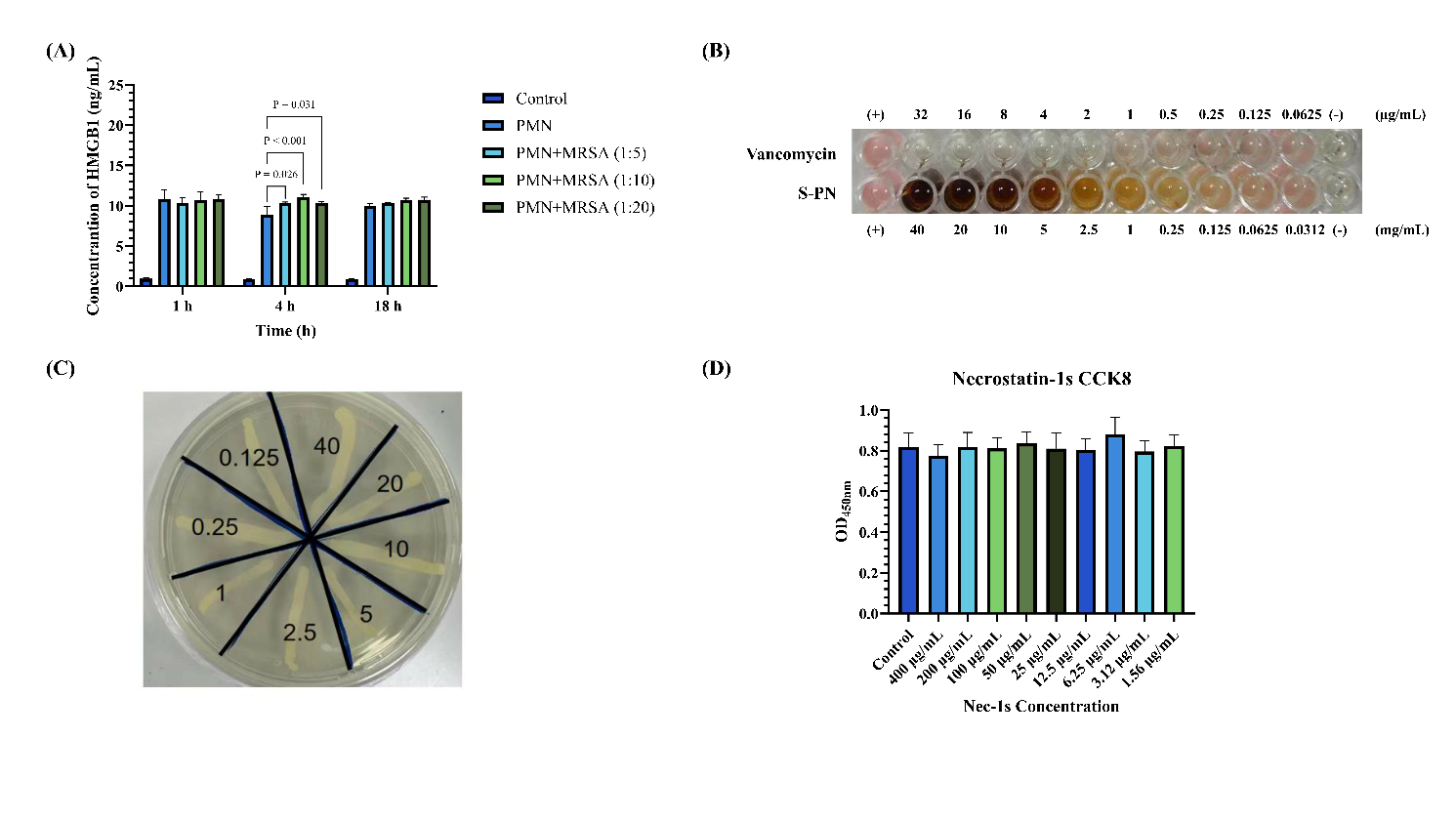


**Supplementary Figure S2. The levels of HMGB1 under different MOI and infection durations interventions and the cytotoxic effects of Nec-1s on PMNs. (**A). Effects of USA300 at different MOI and infection durations on the level of HMGB1 in PMN. Control group refers to the medium-only control, without any additional treatments. Compared with PMN group. (B). The MIC of vancomycin (0.0625–32 μg/mL) and S-PN (0.0312-40 mg/mL) against USA300. (C). The MBC of S-PN (0.125–40 mg/mL) against USA300. (D). Control group represents the PMNs-only control.

# Detection of Granulocyte Count and ROS Levels

Figure S3 present the complete results of three independent biological replicates for both flow cytometric analysis of granulocyte counts and DCFH-DA-based ROS level detection across Control, Model, and S-PN groups. The Model group exhibited a consistent reduction in granulocyte population accompanied by elevated ROS production compared to the Control group, while S-PN treatment effectively reversed these alterations by restoring granulocyte numbers and suppressing ROS accumulation. Notably, all triplicate experiments demonstrated statistically significant intergroup differences with identical trends, complementing the representative flow cytometry images shown in the main text.


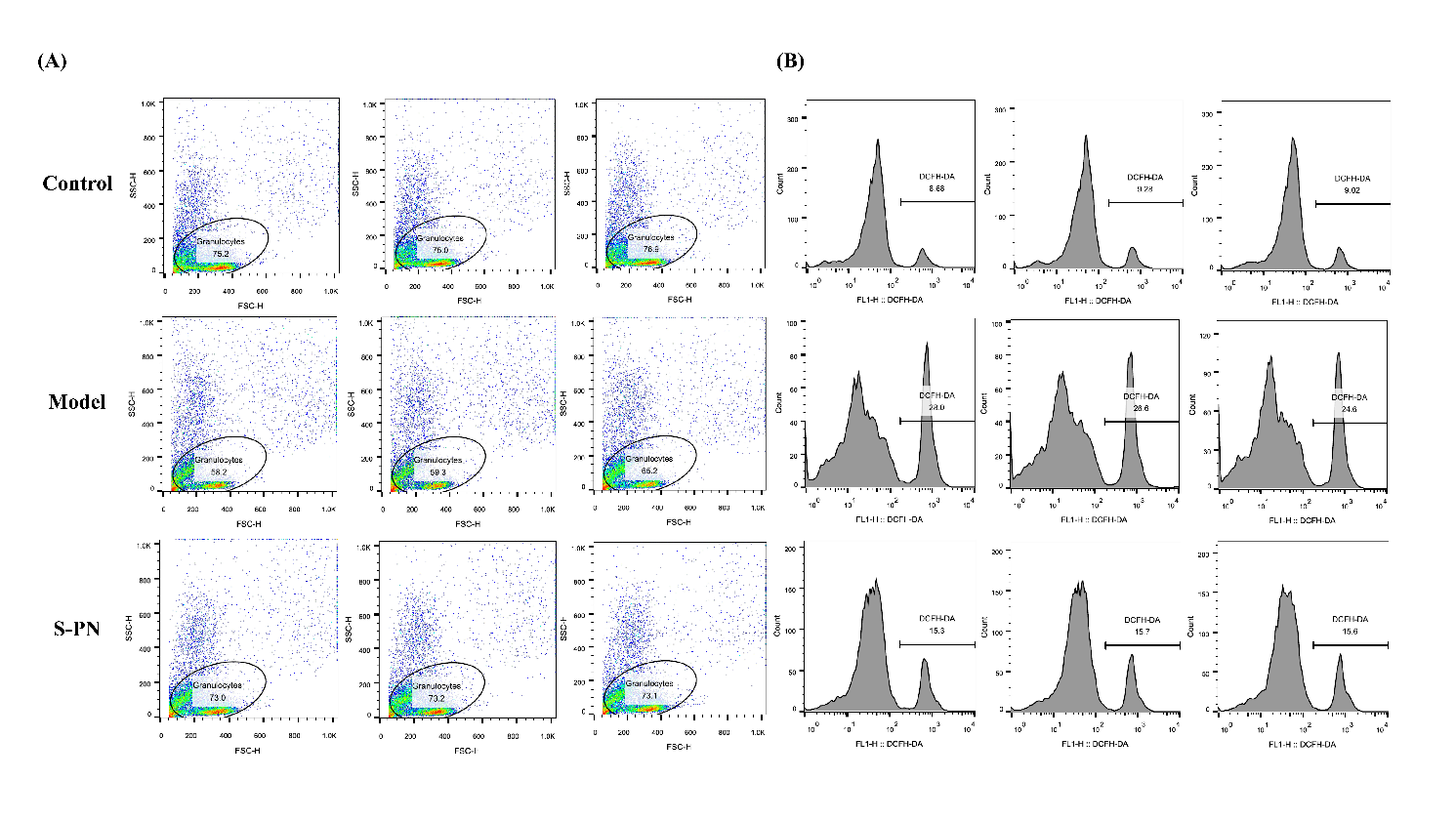


**Supplementary** **Figure S3. The number of granulocytes and ROS levels were measured by flow cytometry.** (A) Flow cytometry analysis of the proportion of total PMNs in each group (n=3). (B) Flow cytometry analysis of ROS levels in each group (n=3).

# Venn, Heatmap and GO enrichment of RNA-seq analysis

Figure S4 provides extended transcriptional profiling data from RNA-seq analysis comparing MRSA (Control group) and S-PN-treated MRSA (S-PN group), including Venn diagrams highlighting shared/unique differentially expressed genes (DEGs), heatmaps depicting clustered expression patterns, and GO enrichment plots visualizing upregulated/downregulated biological pathways.


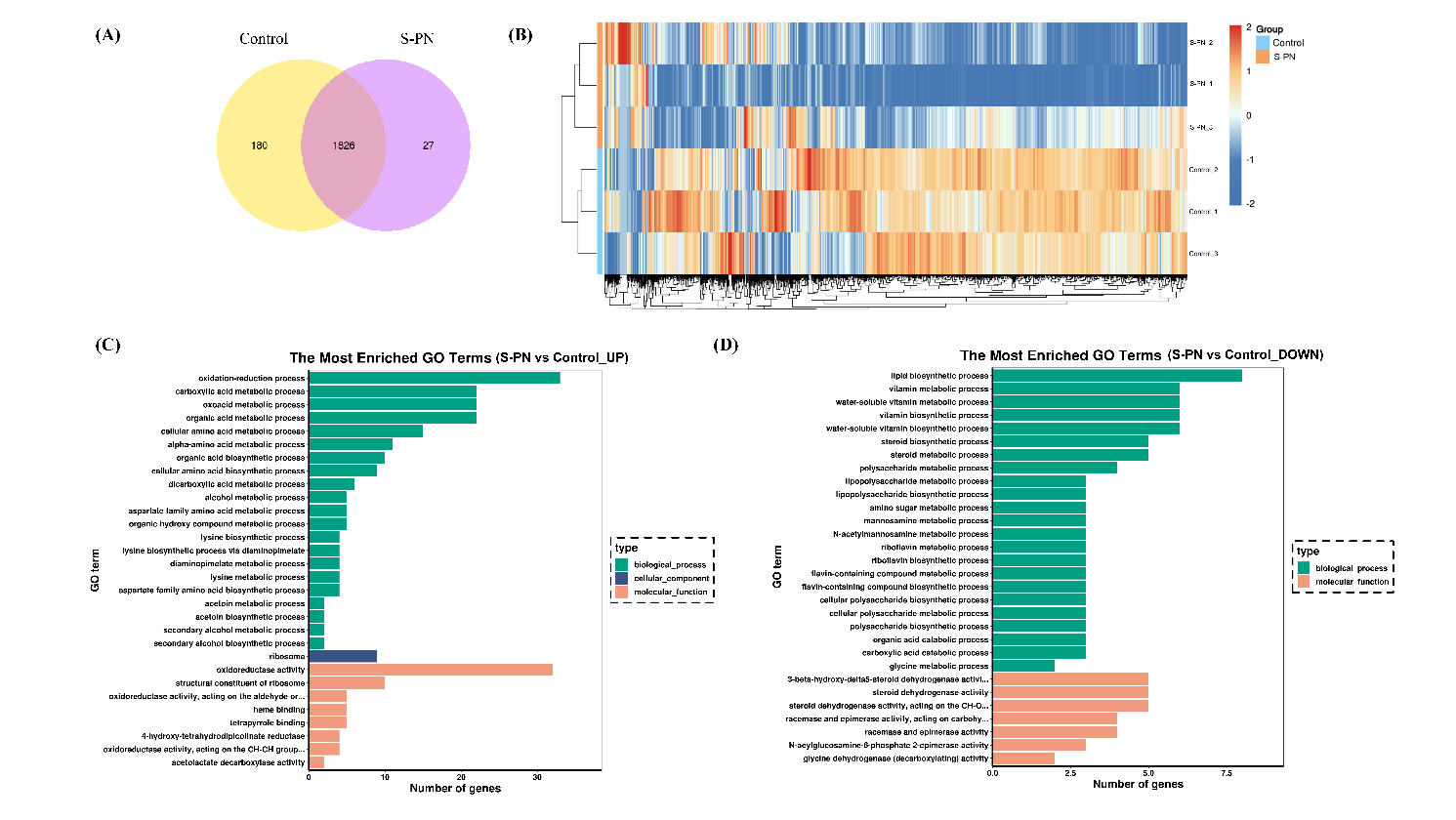


**Supplementary Figure S4. Effect of S-PN on MRSA Gene Transcription.** (A). Venn diagram showing the overlap of MRSA gene transcripts with or without S-PN intervention. (B). Heatmap. S-PN vs Control, with red indicating significantly upregulated genes and blue indicating significantly downregulated genes. (C)-(D). GO enrichment analysis showing significantly upregulated and downregulated pathways.

# DEGs in *Staphylococcus aureus* Infection and Quorum Sensing Pathways

S-PN intervention induced significant changes in the expression of *Staphylococcus aureus* infection and quorum sensing pathways in MRSA, with consistent suppression of enriched DEGs in both pathways (Figure S5).


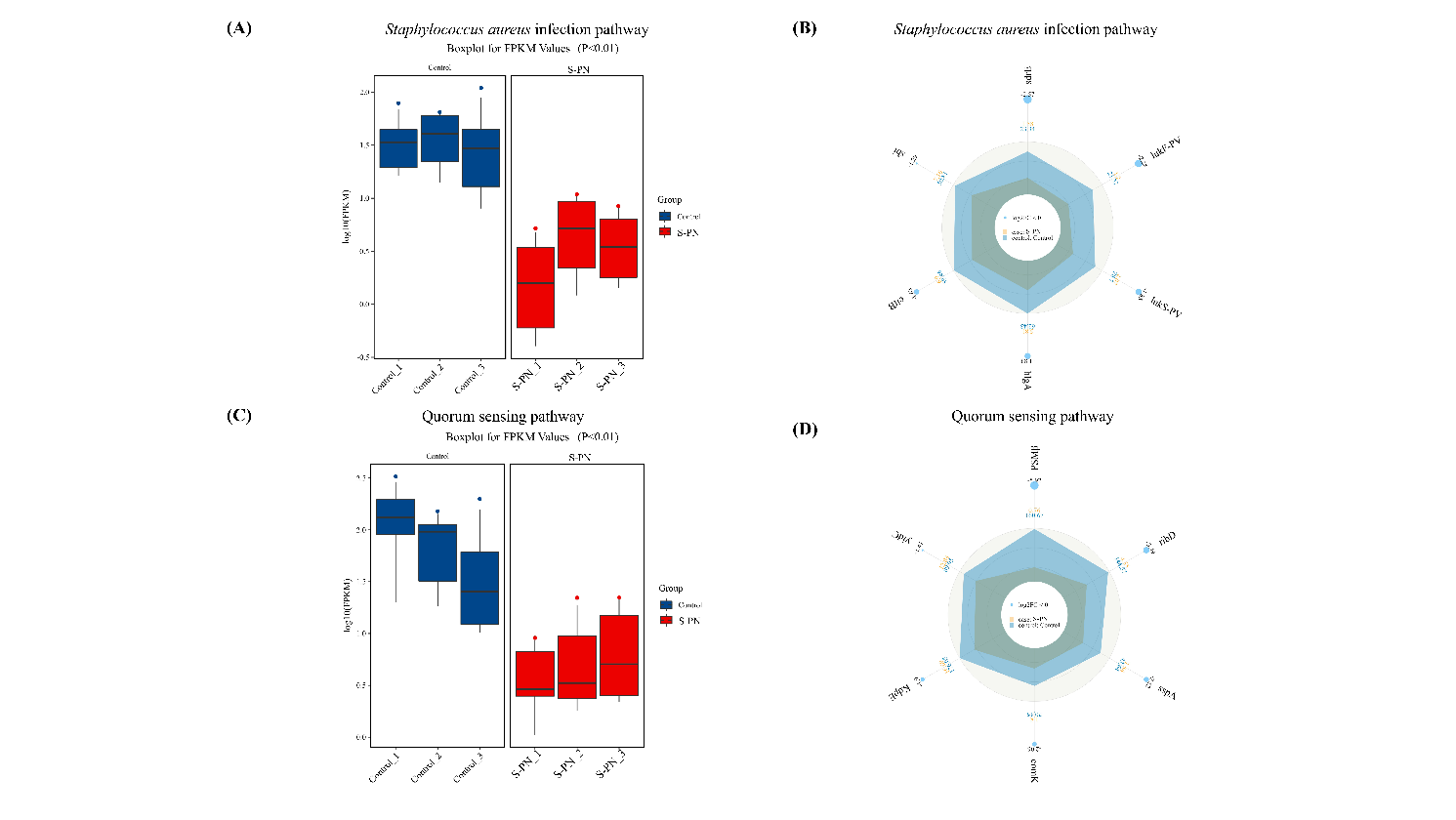


**Supplementary Figure S5. Expression Profiles and Intergroup Differences of Enriched Genes in *Staphylococcus aureus* Infection and Quorum Sensing Pathways.** (A), (C). The boxplot of mean expression of gene in each group in *Staphylococcus aureus* infection pathway and Quorum sensing pathway. (B), (D). Radar chart of hub gene expression levels in *Staphylococcus aureus* infection pathway and Quorum sensing pathway. The outer circle of the plot displays circles whose sizes are proportional to log_2_FC values of the genes, with larger circles representing greater FC. Blue circles indicate downregulated genes. The inner circle represents the average expression levels of genes within each group, providing a visual comparison of overall expression trends across conditions.

# RT-PCR Primer Sequences, RNA Extraction, and Amplification Results

The primer sequences used in this study are shown in Table S2. The electrophoresis results show that the bands of each group are intact and there is no trailing phenomenon, indicating that the extracted RNA have no degradation and can be used for later experiments (Figure S6A-B).


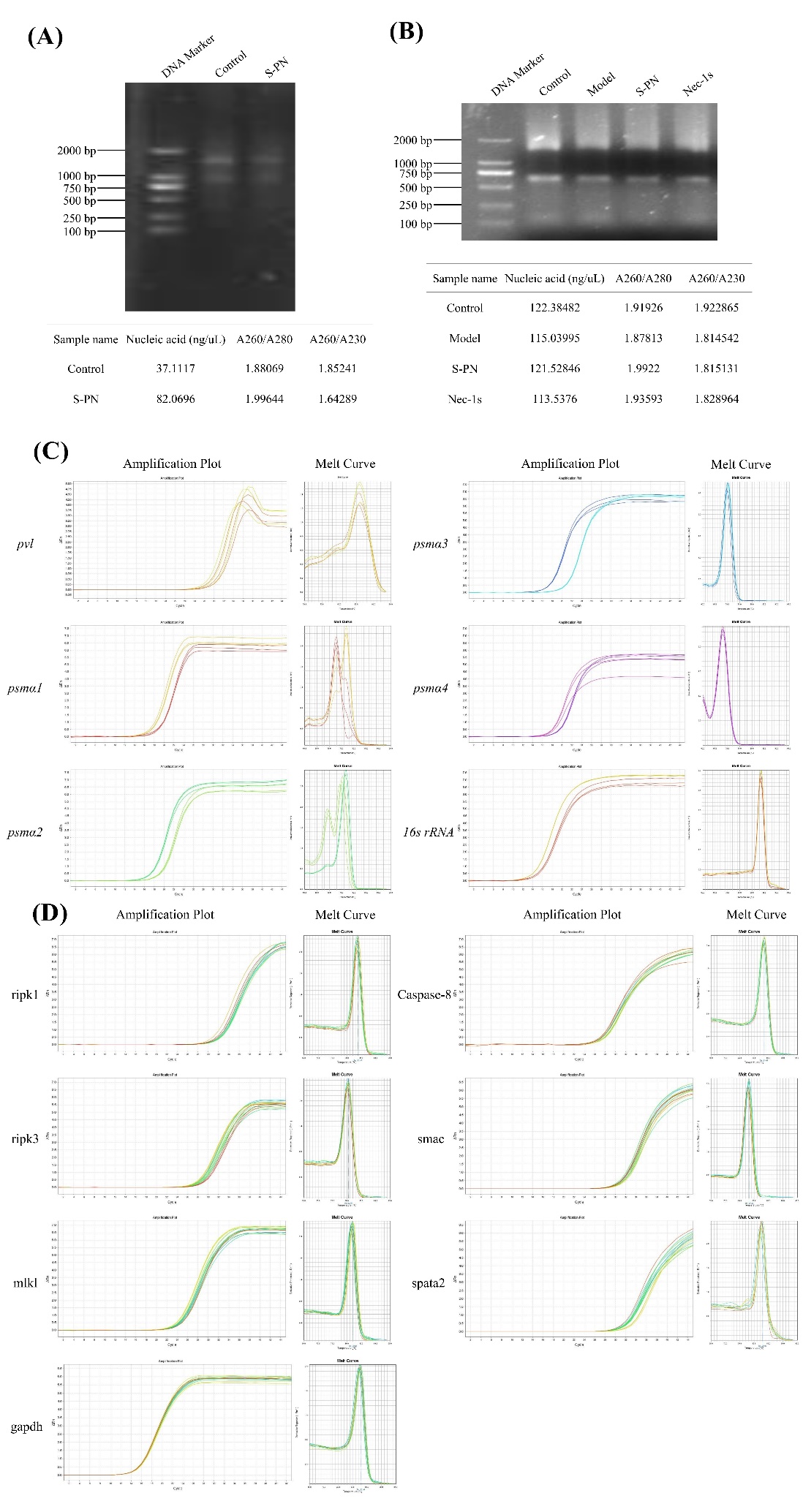


**Supplementary Figure S6. Denaturing agarose gel electrophoresis and amplification and melt curve of RNA.** A. Denaturing gel electrophoresis images of RNA extracted from Control (MRSA) group and S-PN group. B. Denaturing gel electrophoresis images of RNA extracted from Control (PMNs) group, Model (MRSA-PMNs) group, S-PN group and Nec-1s group. C. Amplification and melt curve of *pvl*, *psmα1*, *psmα2*, *psmα3* and *psmα4*. D. Amplification and melt curve of ripk1, ripk3, mlkl, caspase-8, smac, spata2 and gapdh.

**Table S2. Primers used in this study**

| **Primer name** | **Primer sequence (5'to3')** | **Product size (bp)** |
| --- | --- | --- |
| *pvl*-F | GAAATGGCTAGGCAACGAC | 101 |
| *pvl*-R | TCAAACCAGTACGGATCACG |  |
| *psm α1*-F | GGGTATCATCGCTGGCATCATTAAA | 58 |
| *psm α1*-R | ACCAGTGAATTGTTCGATTAAGCTT |  |
| *psm α2*-F | TGGGTATCATTGCAGGAATCATTAA | 62 |
| *psm α2*-R | ACTTACCAGTGAATTTCTCAATTA |  |
| *psm α3*-F | TTCGTAGCAAAATTATTCAAATTCT | 74 |
| *psm α3*-R | AATGTTTGAGATTAGTTGTTACC |  |
| *psm α4*-F | GTACTATCATTAAAATCATCAAAGC | 47 |
| *psm α4*-R | TTTTGCGAAAATGTCGATAATTGCT |  |
| 16s rRNA-F | TTCTGGTCTGTAACTGACGCTG | 299 |
| 16s rRNA-R | CGAAGGGGAAGGCTCTATCT |  |
| ripk1-F | GTCCTGGTTTGCTCCTTC | 123 |
| ripk1-R | TGTTTCGTCTGCCTGTCC |  |
| ripk3-F | GGAGCCAAATCCAGTAAC | 180 |
| ripk3-R | CCTTCTTGCGAACCTACT |  |
| caspase-8-F | CGCAAAGGAAGCAAGAAC | 200 |
| caspase-8-R | TTGAGCCCTGCCTGGTGT |  |
| mlkl-F | TGGAGGAGGCTAATGGGG | 184 |
| mlkl-R | CCTTGGCTTATGGGTGAAA |  |
| smac-F | AACCACTTGGATGACTGC | 185 |
| smac-R | GCTCTTCTATCTGTGCTTCT |  |
| spata2-F | AGAAGCATCAAGACCTACAC | 173 |
| spata2-R | AGCTCAAAGGAGACCATC |  |
| gapdh-F | CACCCACTCCTCCACCTTTGA | 188 |
| gapdh-R | TCTCTCTTCCTCTTGTGCTCTTGC |  |

**Reference**

Fankam, A.G., Kuete1, V., Voukeng, I.K., Kuiate, J.R., and Pages, J.-M. (2011). Antibacterial activities of selected Cameroonian spices and their synergistic effects with antibiotics against multidrug-resistant phenotypes. *BMC complementary alternative medicine* 11**,** 104. doi: 10.1186/1472-6882-11-104.

Yang, W., Liu, J., Blažeković, B., Sun, Y., Ma, S., Ren, C., et al. (2018). In vitro antibacterial effects of Tanreqing injection combined with vancomycin or linezolid against methicillin-resistant Staphylococcus aureus. *BMC Complement Altern Med* 18(1)**,** 169.
